# The JNK signaling pathway in intervertebral disc degeneration

**DOI:** 10.3389/fcell.2024.1423665

**Published:** 2024-09-19

**Authors:** Ganggang Liu, Lu Gao, Yuncai Wang, Xinsheng Xie, Xuejiao Gao, Xingjie Wu

**Affiliations:** ^1^ Orthopaedics, The Second Affiliated Hospital of Heilongjiang University of Chinese Medicine, Harbin, China; ^2^ Otolaryngology, The First Affiliated Hospital of Heilongjiang University of Chinese Medicine, Harbin, China

**Keywords:** intervertebral disc degeneration (IDD), JNK path, programmed cell death (PCD), cellular senescence (CS), cell proliferation, inflammation, extracellar matrix (ECM) degradation, oxidative stress (OS)

## Abstract

Intervertebral disc degeneration (IDD) serves as the underlying pathology for various spinal degenerative conditions and is a primary contributor to low back pain (LBP). Recent studies have revealed a strong correlation between IDD and biological processes such as Programmed Cell Death (PCD), cellular senescence, inflammation, cell proliferation, extracellular matrix (ECM) degradation, and oxidative stress (OS). Of particular interest is the emerging evidence highlighting the significant involvement of the JNK signaling pathway in these fundamental biological processes of IDD. This paper explores the potential mechanisms through the JNK signaling pathway influences IDD in diverse ways. The objective of this article is to offer a fresh perspective and methodology for in-depth investigation into the pathogenesis of IDD by thoroughly examining the interplay between the JNK signaling pathway and IDD. Moreover, this paper summarizes the drugs and natural compounds that alleviate the progression of IDD by regulating the JNK signaling pathway. This paper aims to identify potential therapeutic targets and strategies for IDD treatment, providing valuable insights for clinical application.

## 1 Introduction

Low back pain (LBP) is a significant global public health concern, causing physical discomfort and potential psychological issues like anxiety, depression, and sleep disturbances (Ugwu and Pope, 2023; Rhon et al., 2024). LBP has now emerged as a leading cause of work-related absences, sick leave, and disability worldwide (Wu et al., 2021; Grolier et al., 2023), has become a heavy burden on public health and social economy (Hartvigsen et al., 2018). Approximately 40% of LBP cases are linked to IDD (Luoma et al., 2000). Various factors, including trauma, infection, genetic predisposition, obesity, smoking, aging, and mechanical stress, can trigger molecular, cellular, and tissue alterations in intervertebral discs (IVD), ultimately leading to IDD (Zhang et al., 2024; Silwal et al., 2023; Wang Y. et al., 2020). Recent research has focused on biological treatment options for IDD, such as gene therapy, growth factor therapy, platelet-rich plasma therapy, cell therapy, and tissue engineering implants (Bowles and Setton, 2017; Ekram et al., 2021; Wang S. Z. et al., 2015; Melrose, 2016). However, current treatment strategies primarily target pain relief or pain control rather than reversing IDD at a cellular or molecular level due to a limited understanding of its pathogenesis (Le Maitre et al., 2015; Adams et al., 2010). Therefore, thorough exploration of the disease’s pathogenic mechanisms is crucial for identifying new targets for early diagnosis and treatment of IDD, as well as for guiding effective treatment and rehabilitation efforts.

## 2 Changes in IVD tissue cells of IDD

**FIGURE 1:**
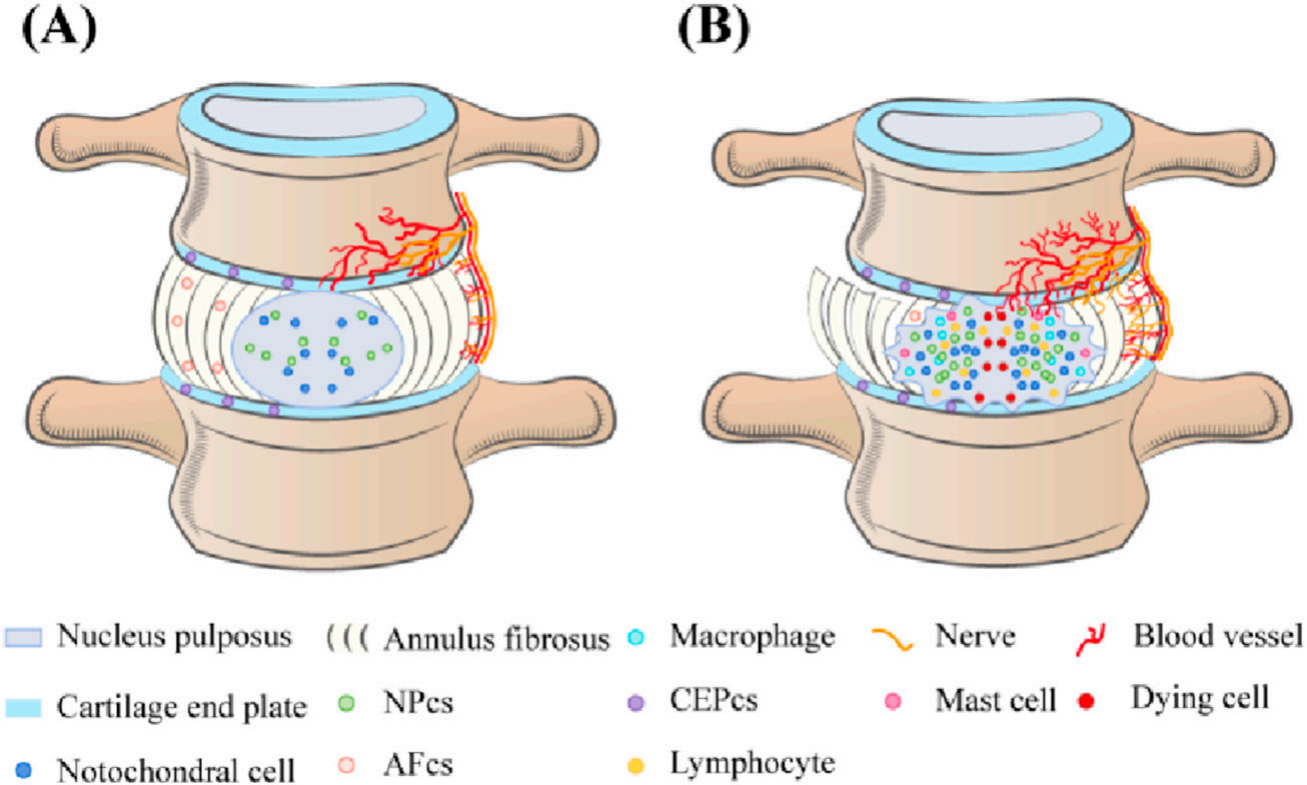
**(A)** In normal IVD, the cell types in NP are notochord cells and NPCs, in AE are AF cells, and in CEP are chondrocytes. Neurovessels exist only in the outer layers of the AF and CEP. **(B)** The number of senescent and dead cells in the degenerated IVD continued to increase, cell density increased, and macrophages, T lymphocytes, and mast cells appeared. The density of microvessels in the endplate decreases, and nerve fibers and microvessels form compensatory growth into the inner layer of AF and even NP tissue. Loss of proteoglycans and water in the NP, reduced IVD height, AF rupture, and destruction or calcification of the CEP.

IVD is composed of highly hydrated gel-shaped nucleus pulposus (NP), annulus fibrosus (AF), and cartilage end plate (CEP) (Kos et al., 2019), playing an important role in maintaining the flexibility and stability of the spine (Sivan et al., 2014). When IDD occurs, there are significant changes in the tissue morphology of the IVD, characterized by the loss of proteoglycans and water in NPs, decreased IVD height, AF rupture, and destruction or calcification of the CEP (Kirnaz et al., 2022; Feng et al., 2017; Edelson and Nathan, 1988). From a cellular perspective, the type, quantity, and function of IVD cells are closely linked to their structural integrity and function. In a healthy IVD, the NP primarily contains notochordal and nucleus pulposus cells (NPCs), while the AF comprises AF cells, and the CEP comprises chondrocytes (Oichi et al., 2020). Notochordal cells disappear from the NP around the age of 10 years (Boos et al., 2002), and their absence is associated with IDD onset (Aguiar et al., 1999; Cappello et al., 2006). IDD may be initiated in early childhood (Yurube et al., 2023). As IDD progresses, the composition of tissue cells within the IVD significantly shifts. Normally, the inner layers of the AF and NP lack blood vessels and nerve tissue, relying on the microvasculature of the adjacent CEP and the surrounding AF for nutrient supply and waste removal (Roberts et al., 2006). However, endothelial cells undergo apoptosis, decreasing endplate microvascular density and impairing nutrient delivery and waste removal in the IVD during IDD (Xu et al., 2019). To compensate for this change, nerve fibers and microvasculature form and grow into the inner layer of the AF, even the NP tissue, forming the anatomical basis for discogenic LBP (Bogduk et al., 1981; Fields et al., 2014; Freemont et al., 2002a). Additionally, immune cells, such as M1 and M2c phenotype macrophages, T lymphocytes, and mast cells, have been detected in degenerative IVD tissues, playing important roles in developing or repairing IVD (Nakazawa et al., 2018; Gorth et al., 2018; Freemont et al., 2002b; Wiet et al., 2017). Notably, a subset of the locally remaining human NPCs generated by a degraded IVD is linked to the development of discogenic pain under specific conditions (Jiang et al., 2023). Although the cell density in the IVD increases during IDD, the number of aging and dead cells continues to rise, accumulating inappropriate metabolites in surviving cells during aging and degradation (Gruber and Hanley, 1998; Hastreiter et al., 2001) (Figure 1). These organizational structures and cell-type alterations are governed by intricate signal transduction networks and interactions with various effector molecules; however, the precise mechanisms remain unclear (Silwal et al., 2023; Vo et al., 2016). Previous studies have indicated that IDD is intricately connected to programmed cell death (PCD), cellular aging, inflammation, proliferation, extracellular matrix (ECM) degradation, oxidative stress (OS), and DNA damage (Zhang et al., 2024; Silwal et al., 2023; Yurube et al., 2023; Cao et al., 2022; Chen et al., 2023).

## 3 Overview of JNK signaling pathways

**FIGURE 2:**
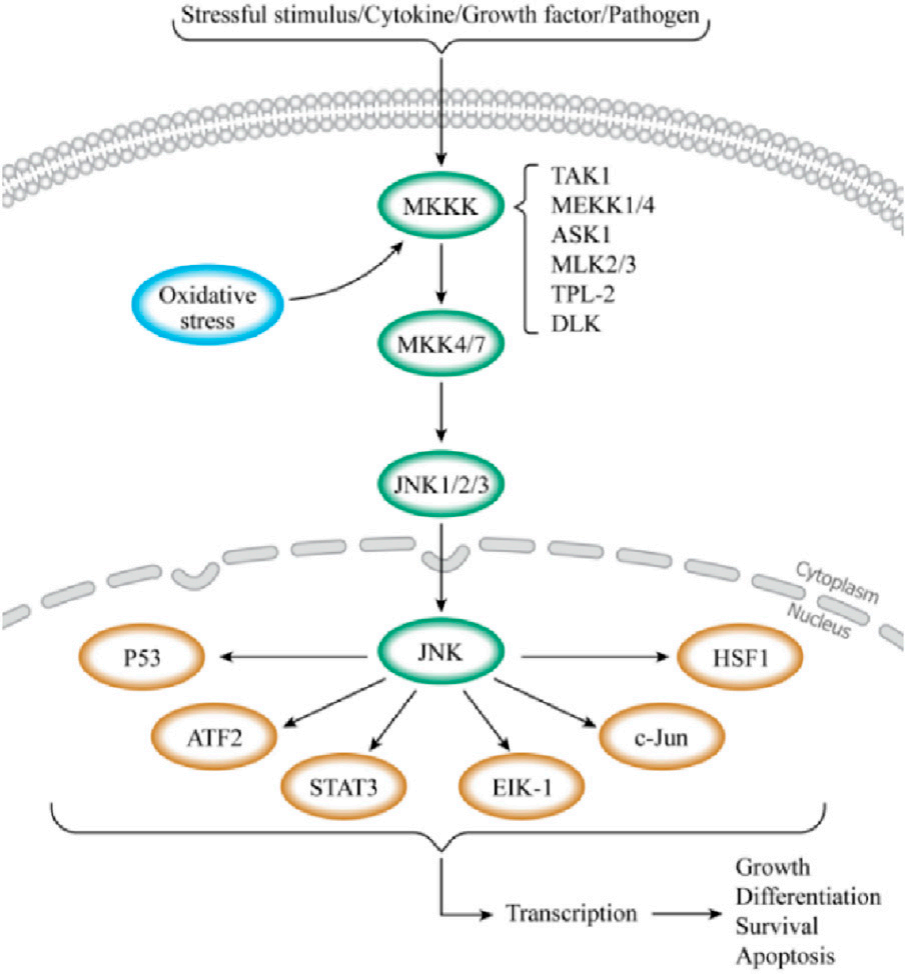
Schematic diagram of the JNK signaling pathway. When cells are stimulated by external stimuli or when receptors on the cell membrane bind to ligands, the JNK signaling pathway is triggered. The activation of this pathway is a three-stage cascade that begins with protein signaling. MAP3Ks (TAK1, MEKK1/4, ASK1, and MLK2/3) are activated in this pathway, further phosphorylating MKK4 or MKK7. JNK is also activated upon phosphorylation by MKK4 or MKK7. Then, activated JNK enters the nucleus, binds to transcription factors such as ATF2, Elk-1, and c-Jun, and regulates the specific gene expression by phosphorylating the active regions of these transcription factors. This process regulates physiological processes, such as cell growth, differentiation, and apoptosis.

c-Jun N-terminal kinase (JNK), also known as stress-activated protein kinase (SAPK), is a key branch of the mitogen-activated protein kinase (MAPK) signaling pathway in mammalian cells (Cui et al., 2007). It plays a significant role in various physiological and pathological (Bogoyevitch and Kobe, 2006; Weston and Davis, 2007). Similar to other MAPK pathways, the JNK pathway is composed of MAP3K (TAK1, MEKK1/4, ASK1, and MLK2/3), MKK4, MKK7, and JNK1/2/3 (Kyriakis et al., 1994; Zeke et al., 2016). Various extracellular stimuli, such as stresses (ultraviolet (UV), mechanical stress, and protein synthesis inhibitors), cytokines [tumor necrosis factor-alpha (TNF-α) and interleukin-beta (IL-1β)], growth factors, pathogens, hormones, and intracellular stressors (OS and DNA damage),extracellular-derived stress signals are introduced into the cell via the corresponding membrane receptors on the cell membrane and activate the JNK signaling pathway (Minden and Karin, 1997; Ichijo, 1999; Monaghan et al., 2014; Wu et al., 2020; Arthur and Ley, 2013; Hsu et al., 2010). Upon stimulation, MAP3K is activated, leading to the phosphorylation of MKK4 or MKK7. Ultimately, the dual phosphorylation functional region, Thr-Pro-Tyr, of JNK is double phosphorylated by MKK4 or MKK7, activating JNK (Bogoyevitch and Kobe, 2006; Zeke et al., 2016; Yan et al., 2024). This series of phosphorylation events forms a cascade reaction for signal amplification (Figure 2). In this process, MKK7 specifically activates JNK, while MKK4 can simultaneously activate both JNK1 and p38 (Haeusgen et al., 2011; Vander et al., 2005). JNK1/2/3 exhibits high sequence conservation throughout evolution (Gupta et al., 1996; Ait-Hamlat et al., 2020), with JNK1 and JNK2 broadly expressed, and JNK3 predominantly found in the brain, heart, testes, and pancreas (Kyriakis et al., 1994; Xie et al., 1998; Garg et al., 2021; Nakano et al., 2020; Vallerie and Hotamisligil, 2010). Activated JNK enters the nucleus and binds to transcription factors, such as ATF2, Elk-1, and c-Jun, phosphorylating their active regions to regulate specific gene expression (Kyriakis et al., 1994; Barr and Bogoyevitch, 2001). JNK can also phosphorylate non-nuclear proteins, such as mitochondrial BCL-2 family members (BCL-XL and BCL-2) (Kyriakis et al., 1994). Besides the core mechanisms mentioned above, the JNK signaling pathway interacts intricately with other pathways, including PI3K/Akt and NF-ĸB, to influence cell growth, survival, and metabolism (Li S. T. et al., 2018; Dong et al., 2022; Kriehuber et al., 2005). JNK activation and regulation are highly complex, with signal transduction dysfunction playing a crucial role in human diseases. Once the JNK pathway is activated, it will transmit signals by phosphorylating a series of substrates, thus affecting the biological processes of IVD cells such as survival, proliferation, differentiation, and apoptosis. Studies have indicated the close relationship between the JNK signaling pathway and processes like PCD, cell aging, inflammation, proliferation, ECM degradation, and OS (Sui et al., 2014; Papaconstantinou, 2019; Pinal et al., 2019; Du X. F. et al., 2023; Levada et al., 2018). Delving into the connection between the JNK signaling pathway and IDD is crucial for uncovering the pathological mechanisms of IDD and identifying new treatment methods and drug targets. This provides new perspectives for IDD treatment and a valuable reference for managing related diseases.

## 4 JNK signaling pathway and IDD

**FIGURE 3:**
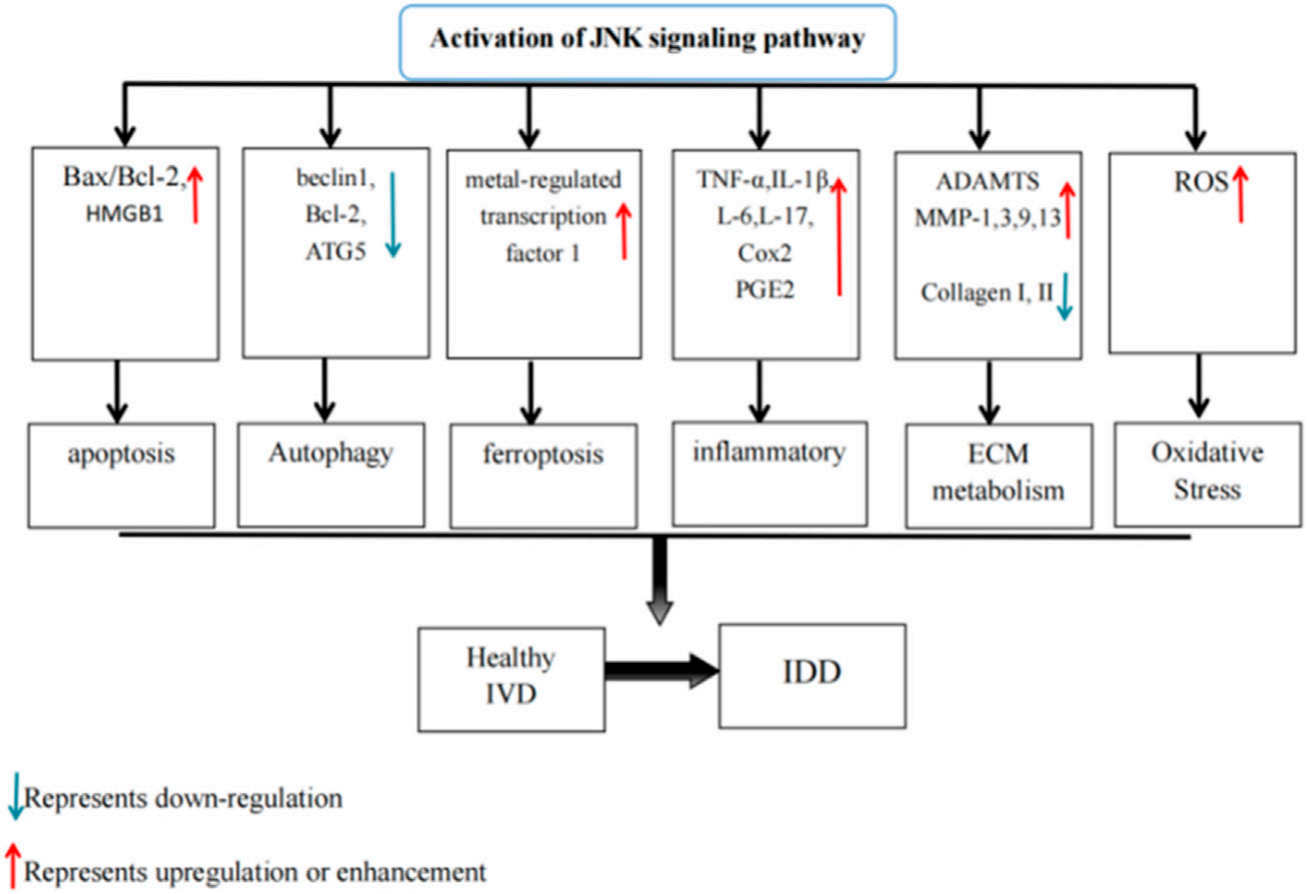
The activation of JNK signaling pathway resulting in changes of various factors in IVD and eventually disc degeneration.

Extracellular factors, such as mechanical stress, inflammation, and infection, as well as intracellular factors, such as DNA damage, can trigger IDD and activate the JNK signaling pathway. Additionally, biological processes linked to JNK, such as cell death, aging, inflammation, proliferation, ECM breakdown, and OS, are well-documented contributors to IDD. Therefore, we have ample reason to believe a close and profound relationship exists between IDD and the JNK signaling pathway. This provides strong theoretical support and practical guidance to further investigate the association between the JNK signaling pathway and IDD (As is shown in Figure 3).

### 4.1 Mediating PCD

Currently, PCD forms such as cell apoptosis, necroptosis, autophagy, pyroptosis, ferroptosis, and PANoptosis have received extensive research attention (Liu et al., 2022). Numerous studies have confirmed that PCD plays a crucial role in the occurrence of IDD (Zhao et al., 2006). Although PANoptosis is a form of PCD, there is currently no literature indicating a direct relationship between it and IDD. However, a relationship between the JNK pathway, cell necroptosis, and pyroptosis has been revealed (Du J. et al., 2023; Liu et al., 2020). Moreover, cell necroptosis and pyroptosis can cause IDD (Khaleque et al., 2023; Luo et al., 2022). However, there is currently insufficient evidence to support whether the JNK pathway is involved in the specific mechanisms of necroptosis and pyroptosis in IDD. Therefore, the mechanism of action of the JNK pathway in necroptosis and pyroptosis in IDD remains unclear, and further research is needed.

#### 4.1.1 Mediating apoptosis of IVD cells

Apoptosis is a key factor leading to a decreased number of cells in NP tissues, and this process plays an important role in degrading IVD (Ding et al., 2013). The JNK signaling pathway is closely linked to cell apoptosis, as confirmed by research (Farrell et al., 2022; Zhou et al., 2022). Recent studies have found that RASSF7 can negatively regulate apoptotic JNK signaling by inhibiting the activity of phosphorylated MKK7 (Takahashi et al., 2011). In human cadaver studies, the RASSF7 expression level was significantly higher in non-degenerative intervertebral disc tissues than in degenerative intervertebral disc tissues (Liu et al., 2015). This discovery suggests that there may be a direct or indirect association between the activation of the JNK signaling pathway and apoptosis of NPCs, and RASSF7 may serve as an inhibitor of the JNK signaling pathway, providing new ideas for treating IDD. Direct evidence suggests that the JNK pathway can mediate the occurrence of IVD via mechanisms such as infection and high osmotic stress. Researchers used animal models infected with *Propionibacterium acnes* to study and found that it induces apoptosis in NPCs via the Toll-like receptor 2/JNK pathway and mitochondrial-mediated cell death mechanism (Lin et al., 2018). Additionally, animal experiments have shown that signal-regulated kinase-1 facilitated the senescence and apoptosis of NP cells in promoting IDD progression via the JNK/p38 pathway (Zou et al., 2024). Bai et al. showed that by modulating the JNK and p38 MAPK signaling pathways, Baicalin attenuates IL-1β-induced apoptosis, oxidative stress, inflammation, and extracellular matrix degradation in nucleus pulposus cells (Bai et al., 2023). Moreover, the JNK signaling pathway is involved in IVD cell apoptosis caused by inflammation. Research has found that TNF-α binding to TNF receptors can activate JNK/ERK-MAPK and NF-κB signaling pathway in NPCs induces inflammation-induced cell apoptosis (Zhang et al., 2019). Meanwhile, rhTSG-6 can protect NPCs from IL-1β-induced apoptosis and ECM degradation by inhibiting the p38, JNK, and ERK pathways (Pei et al., 2020). The JNK signaling pathway also involves other molecular mechanisms linked to IVD cell apoptosis. For example, glycyrrhizin can inhibit the expression of high mobility group protein box1 (HMGB1) via the p38/p-JNK signaling pathway, thereby reducing inflammation and cell apoptosis in human NP (Liu et al., 2019). In summary, there is a close correlation between the JNK pathway and apoptosis in IVD cells. Excessive JNK activation in IVD cells is the main driving factor for IVD cell apoptosis, reduced ECM production, and IDD progression. Therefore, in-depth research on the mechanism of the JNK pathway in IDD is of great significance for revealing the pathological process of IDD and for developing new treatment strategies.

#### 4.1.2 Regulating autophagy in IVD cells

Autophagy is significantly enhanced in degenerative IVD cells than in normal IVD cells (Ye et al., 2013). Xu et al. (2015) found that inhibition of NF-κB and JNK pathways can enhance autophagy in NPCs of rat. Research has found that overexpression of islet amyloid polypeptide upregulates the phosphorylation of AKT and mTOR while downregulating the expression of phosphorylated p38 and JNK, as well as downregulating the gene expression such as Bcl-2, Beclin-1, and ATG5, increasing autophagy and reducing apoptosis in NPCs (Wu et al., 2017). Li Z. et al. (2021) revealed that compressive stress induces NPCs autophagy by regulating the PI3K/AKT/mTOR pathway associated with reactive oxygen species (ROS) and activating the JNK pathway. JNK pathway inhibition caused cell death and increased ROS generation, decreasing NP cell autophagy capacity. Additionally, Zhang Y. et al. (2023) conducted experiments on large-eared white female rabbits and discovered the tension load-induced cartilage endplate stem cell (CESC) degeneration model, inhibiting the expression of JNK and ERK can inhibit the phosphorylation of Raptor and mTOR in the mTOR signaling pathway, thereby increasing the autophagy level of CESC and alleviating its degradation. In Berberine’s research on preventing OS-induced cell apoptosis, the IRE1/JNK pathway induced endoplasmic reticulum stress-dependent autophagy (Luo et al., 2019). This evidence indicates that the JNK pathway plays a complex and important regulatory role in autophagy in IVD cells.

#### 4.1.3 Cell ferroptosis involved in IDD

Ferroptosis plays an important role in IDD (Wang et al., 2022). The JNK signaling pathway is crucial for ferroptosis-induced degenerative lesions (Sun et al., 2023). Lu et al. found that (Lu et al., 2021) Hinokitiol enhances the nuclear translocation of metal-regulated transcription factor 1 by inhibiting the JNK pathway, restoring the normal function of FPN, reducing intercellular iron overload under OS, protecting human NPCs from iron death, and improving the progression of IDD *in vivo*. This indicates that the JNK signaling pathway plays a role in the ferroptosis mechanism of IVD cells. However, further research is needed to elucidate its mechanism due to the limited research on the involvement of the JNK signaling pathway in the ferroptosis mechanism of IVD cells.

### 4.2 Inducing inflammatory response

The hallmarks of IDD are ECM degradation, apoptosis, and inflammation (Cazzanelli and Wuertz-Kozak, 2020). Multiple pro-inflammatory cytokines, such as TNF and IL-1β. Increased IL-6 and IL-17 levels cause IVD tissue degeneration and pain induction (Risbud and Shapiro, 2014). Evidence suggests that the JNK signaling pathway plays a crucial role in the increased expression of inflammatory factors in IVD. Specifically, IL-17A activates the JNK and P38-MAPK signaling pathways, increasing COX2 expression and PGE2 production in an activating protein-1 -dependent manner and mediating IVD inflammation (Li J. K. et al., 2016). Additionally, Visfatin upregulates IL-6 expression in NPCs by activating the JNK/ERK/p38-MAPK signaling pathway (Cui et al., 2021). IL-1β-induced inflammatory response and pain in IVD cells (Li W. et al., 2015). Tanshinone IIA regulates the expression of interleukin-1 receptor-associated kinase 1, p38, JNK, and NF-κB signaling pathway to effectively inhibit inflammatory response and pain in IVD cells. Additionally, studies have shown that piperine inhibits lipopolysaccharide (LPS) mediated JNK phosphorylation, and NF-κB activation significantly reduced IL-1β and TNF-α. The expression of various inflammatory factors, such as IL-6 and OS-related genes (Li Y. et al., 2015). The JNK signaling pathway also participates in the inflammatory process of IVD via other pathways. Sesamin and Carthamin yellow effectively inhibited NP cell matrix degradation and inflammation induced by LPS via the MAPK signaling pathway by inhibiting the JNK/P38/ERK phosphorylation (Chen et al., 2017; Li K et al., 2016). Acacetin enhances antioxidant protein expression, such as HO-1, NQO1, and SOD, by activating the Nrf2 pathway and inhibiting phosphorylation of p38, JNK, and ERK1/2, thereby improving inflammation and matrix degradation in NPCs (Wang H. et al., 2020). JNK signaling pathways cross-interact with other signaling pathways, jointly participating in the inflammatory process in IDD. Animal experiments have shown that the JNK signaling pathway plays an important role in Wnt5a’s inhibition of inflammation, leading to IDD via the TNF-α/NF-κB Wnt5a negative feedback loop (Li Z. et al., 2018). Chen et al. (2021) found that Dehydroostus lactone can inhibit the activation of JNK/P38 mapk/ERK and NF-κB inflammatory signaling pathway, and improve the aging of NPCs activated by the STING-TBK1/NF-κB signaling pathway. Besides, the JNK signaling pathway can exert their effect in the inflammatory response in IVD induced by pro-inflammatory macrophages. Inhibiting the JNK pathway can reduce metabolic and inflammatory gene expression induced by M1 polarized RAW264.7 cells (Ni et al., 2019). In summary, the JNK signaling pathway plays a crucial role in inflammatory factor expression and inflammatory processes in IVD. Comprehensively studying its potential therapeutic targets and implementing targeted interventions is an important direction for alleviating IDD and treating discogenic pain.

### 4.3 Regulating ECM metabolism

ECM degradation, an important criterion for IDD, has always been a hot research topic regarding its regulatory mechanism (Cazzanelli and Wuertz-Kozak, 2020). Specific stimuli or substances play a crucial role in ECM metabolism in IVD cells by regulating the JNK pathway activity. The JNK signaling pathway independently regulates ECM metabolism in IVD cells and interacts with other signaling pathways to regulate the metabolic balance of the ECM. Research has indicated that under hypoxic conditions, the JNK signaling pathway is involved in the ECM degradation of NPCs induced by serum deprivation by activating JNK1/2 (Wang et al., 2017). Additionally, Rhizoma drynariae total flavonoids were extracted from Drynaria fortunei J. Sm reduced the matrix metalloproteins expression by inhibiting the JNK/P38-MAPK/ERK signaling pathway and regulating the expression of aggregates and collagen types I, II, and III, thereby effectively inhibiting ECM degradation. This discovery reveals the potential of traditional Chinese medicine in treating ECM degradation by regulating the JNK signaling pathway (Zhao et al., 2021). The JNK signaling pathway can interact with other pathways to regulate ECM metabolism. For example, FAM20B regulates TGF-β. The JNK/P38-MAPK/ERK signaling pathway affects glycosaminoglycans synthesis, thereby regulating ECM metabolism and cell degeneration in AF cells (Saiyin et al., 2019). Additionally, platelet-driven growth factor-BB promotes ECM metabolism in NPCs by regulating the ERK, JNK, and PI3K/AKT signaling pathways while inhibiting pyroptosis and the production of pyroptosis products in NPCs (Zhang et al., 2022). The JNK signaling pathway also promotes an imbalance in extracellular matrix metabolism in NPCs by M2a macrophages (Li L. et al., 2021). Therefore, the JNK signaling pathway is crucial in regulating ECM metabolism.

### 4.4 Accelerate IVD cell aging

Cellular senescence (CS) is a key factor in aging, and aging cells accumulate continuously with age (Roger et al., 2021). Aging has been proven to be the primary cause of IDD, and CS is an important mechanism for IDD occurrence (Silwal et al., 2023). During this process, the JNK signaling pathway plays a crucial role in CS (Papaconstantinou, 2019; Grootaert et al., 2021). Further research has indicated that the JNK signaling pathway plays an important role in the aging of IVD cells. Specifically, stress can activate the cascade response of the JNK signaling pathway, thereby participating in stress-induced premature aging of IVD cells (Patil et al., 2018; Maruyama et al., 2009). Additionally, studies have shown that activation of heat shock protein 70 can inhibit the JNK/c-Jun pathway and hinder apoptosis and aging of human NP stem cells induced by tert-butyl hydroperoxide (Zhang et al., 2021). However, the mechanism by which the JNK signaling pathway regulates intervertebral disc cell aging remains unclear due to relatively limited research. Therefore, more in-depth research is needed to explore this issue and to confirm whether the JNK signaling pathway may be a direct therapeutic target for reducing IVD cell aging. This will provide new ideas and directions for treating IDD and relieving discogenic pain.

### 4.5 Regulation of IVD cell proliferation

The normal physiological function of IVD tissue is established based on healthy cell proliferation and tissue repair ability (Bhujel et al., 2022). The close relationship between the JNK signaling pathway and cell proliferation has attracted attention during this process (Wang JX et al., 2023). Previous research has shown that the JNK signaling pathway plays an important regulatory role in IVD cell proliferation, and its abnormal activation may inhibit IVD cell proliferation, thereby accelerating IDD development. Dimozi et al. (2015) confirmed this by finding that the p38-MAPK, ERK, and JNK signaling pathways are involved in H_2_O_2_-induced G1 cell cycle delay and reduced cell proliferation in NPCs. Additionally, TNF-α, an important inflammatory factor, is NF-κB. The JNK and p38-MAPK pathways regulate the proliferation of NPCs (Wang X. H. et al., 2015). The JNK signaling pathway plays a crucial role in regulating IVD cell proliferation, providing new perspectives and ideas to understand the pathogenesis of IDD at the molecular level.

### 4.6 Participate in OS in IVD cells

ROS and OS are closely related to the occurrence of IDD (Feng et al., 2017). Excessive ROS generation triggers the OS response of IVD cells (Suzuki et al., 2015). The JNK signaling pathway is involved in ROS accumulation in IVD cells during this process. In the rat tailbone IDD model, andrographolide upregulated the expression of heme oxygenase-1 (HO-1), p-Nrf2, p-p38, p-Erk1/2, and p-JNK, activating the MAPK/Nrf2/HO-1 signaling pathway and effectively inhibiting static mechanical pressure-induced apoptosis and ROS accumulation in NPC (Zhang C. et al., 2023). Additionally, the JNK signaling pathway plays an important role in the OS response of IVD cells. Research has shown that the JNK/P-38/ERK signaling pathway is involved in mediating the inhibitory effect of amobarbital and N-acetylcysteine on OS in NPCs (Seol et al., 2021). Bone marrow mesenchymal stem cells rapidly promote mitophagy via the JNK signaling pathway in the early stages of IDD OS. However, they reduce mitophagy and increase apoptosis in the later stages, demonstrating their complex regulatory mechanisms in response to OS (Fan et al., 2019). Although there is currently relatively limited research on the JNK signaling pathway in OS in IVD cells, it can be concluded that the JNK signaling pathway plays an irreplaceable role in the OS mechanism in IVD cells.

### 4.7 Multiple pathogenic mechanisms involved in IDD

The JNK signaling pathway plays a crucial role in the pathogenesis of IDD and simultaneously participates in multiple regulatory processes. Tang et al. (2018) found that Honoriol inhibits the XNIP/NLRP3/caspase-1/interleukin-1 signaling axis by effectively blocking the activation of NF-kB and JNK, thereby mitigating H_2_O_2_-induced apoptosis, OS, and inflammatory responses in NPCs. Similarly, Chen et al. (2020) found that quinazoline dephosphorylates key signaling molecules, such as IKKβ, IκBα, and NFs, such as p65, ERK, JNK, and p38, thereby inhibiting IL-1β-induced OS and inflammation via the NF-κB/MAPKs signaling pathway, promoting cell proliferation, and preventing NP cell degeneration. Since IL-1β can activate p38 MAPK, c-Jun, and NF-κB signaling pathways, leading to IVD degeneration, inhibiting the IL-1β signaling pathway is crucial in preventing IDD progression (Daniels et al., 2017). Wang et al. conducted Network pharmacological analysis and revealed that the active compound Kaempferol in *Eucommia ulmoides* significantly reduces IL-1β levels and promotes phosphorylation of p38, JNK, and ERK1/2, thereby protecting NPCs from IL-1β-induced damage via multiple mechanisms (Wang X. et al., 2023). Furthermore, the JNK signaling pathway regulates IDD via various pathways. Reports indicate that high-temperature requirement A1 regulates ADAMTS expression in human NPCs via the JNK pathway, thereby contributing to IDD development (Li et al., 2019). Syndecan-4 also influences NP cell function and IDD progression by activating the JNK and p53 signaling pathways (Ge et al., 2020). Leptin upregulates catabolic gene expression in rat NPCs by modulating the JNK/P38-MAPK/ERK and JAK2/STAT3 pathways (Miao and Zhang, 2015). Hu et al. (2018) found that TGF-β stimulates the expression of chondroitin polymerizing factor and sulfated glycosaminoglycan in NPCs via various signaling pathways, including Smad3, RhoA/ROCK1, JNK, p38, and ERK1/2. This highlights the potential therapeutic role of TGF-β in IDD.

From the foregoing, it can be inferred that in the process of IDD, apoptosis, OS, inflammation, and ECM degradation may occurr at the same time; And apoptosis and cell aging may occurr at the same time. However, whether these processes occur in parallel or simultaneously when IDD is present, is not supported by evidence and further research is needed to confirm.

## 5 Drugs and natural compounds targeting the JNK signaling pathway

As summarized above, the JNK signaling pathway plays a crucial role in the pathological process of IDD. Research on drugs and natural compounds that alleviate the progression of IDD by regulating the JNK signaling pathway is of great significance. However, according to literature searches, there are currently very limited reports on how drugs and natural compounds influence the progression of IDD through the regulation of the JNK pathway. Only 19 drugs or natural compounds that can regulate the JNK pathway to affect the progression of IDD have been identified in published literature (Regulation mechanism of the JNK pathway and research conclusions are shown in Table 1). Therefore, there is broad potential and significant room for exploration in the treatment of IDD with drugs and natural compounds targeting the JNK signaling pathway. We should enhance the development and research of clinical drugs targeting the JNK pathway and build evidence-based support based on big data to further promote its application and development in the field of IDD treatment.

**TABLE 1:** Drugs and natural compounds targeting the JNK signaling pathway.

Classification	Drugs And natural Compounds	Regulation mechanism of JNK pathway	Research conclusion	Author and citation
Drugs	Du Zhong	Reduce the promoting effect of IL-1β stimulation on p38, JNK and ERK1/2 phosphorylation	Protect NP cells from multiple mechanisms of damage induced by IL-1β	Wang J. X. et al. (2023)
	Amobarbital	Inhibit tert-butyl hydrogen peroxide-induced JNK and p38 MAPK activation	Improve mitochondrial function, reduce apoptosis, necrosis, and ROS production in IVD cells	Seol et al. (2021)
	Amobarbital	Suppressing the NF-kB and MAPK Pathways in Nucleus Pulposus Cells	Inhibits IL-1β-Induced Apoptosis and Extracellular Matrix Degradation	Tu et al. (2017)
Natural compounds	Baicalin	modulating the JNK and p38 MAPK signaling pathways	Inhibition of IL-1β-induced NP cell apoptosis, oxidative stress, inflammation and extracellular matrix degradation	Bai et al. (2023)
	Glycyrrhizin	Inhibition of HMGB1 expression through p38/p-JNK signaling pathway	Reduce inflammation and apoptosis in human NP	Liu et al. (2019)
	Berberine	The IRE1/JNK pathway is involved in the induction of endoplasmic reticulum stress-dependent autophagy	Prevent oxidative stress-induced apoptosis	Luo et al. (2019)
	Hinokitiol	Inhibit the JNK pathway to enhance the nuclear translocation of metal regulatory transcription factor 1 (MTF1)	Restore the normal function of FPN, reduce intracellular iron overload under oxidative stress, and protect human NP cells from ferroptosis	Lu et al. (2021)
	Anshinone IIA	Regulates the expression of interleukin-1 receptor-associated kinase 1 (IRAK-1) and the p38, JNK and NF-κB signaling pathways	Inhibit IL-1β-induced inflammatory response and induced pain in IVD cells	Li Y. et al. (2015)
	Piperine	Inhibit lipopolysaccharide (LPS)-mediated JNK phosphorylation and NF-κB activation	Reduce the expression of multiple inflammatory factors such as IL-1β, TNF-α, IL-6, and oxidative stress-related genes	Li K. et al. (2015)
	Carthamin yellow	Activate Nrf2 pathway and inhibit p38, JNK and ERK1/2 phosphorylation	Increase the expression of antioxidant proteins such as HO-1, NQO1 and SOD, and improve the inflammation and matrix degradation of np cells	Chen et al. (2017)
	Sesamin	Activate Nrf2 pathway and inhibit p38, JNK and ERK1/2 phosphorylation	Increase the expression of antioxidant proteins such as HO-1, NQO1 and SOD, and improve the inflammation and matrix degradation of np cells	Li J. K. et al. (2016)
	Acacetin	Activate the Nrf2 pathway and inhibit p38, JNK, and ERK1/2 phosphorylation, increasing the expression of antioxidant proteins such as HO-1, NQO1, and SOD	Improve the inflammation and matrix degradation in NP cells	Wang Y. et al. (2020)
	Dehydrocostus lactone	Inhibits the activation of JNK/P38-mapk/ERK and NF-κB inflammatory signaling pathways	Improve the aging of NP cells caused by activation of STING-TBK1/NF-κB signal	Chen et al. (2021)
	Rhizoma drynariae total flavonoids (RDTF) extracted from Drynaria fortunei J. Sm (D. fortunei)	Inhibiting the JNK/P38-MAPK/ERK signaling pathway reduces the expression of matrix metalloproteinases (MMPs) and regulates the expression of aggrecan and collagen type I, II, and III	Inhibit the degradation of ECM	Zhao et al. (2021)
	Andrographolide	Upregulates the expression of Heme oxygenase-1 (HO-1), p-Nrf2, p-p38, p-Erk1/2, and p-JNK, and activates the MAPK/Nrf2/HO-1 signaling pathway	Inhibit NPC Static mechanical stress induces apoptosis and ROS accumulation	Zhang C et al. (2023)
	Honokiol	Inhibit the XNIP/NLRP3/caspase-1/interleukin-1β signaling axis and the activation of NF-kB and JNK	Inhibit H_2_O_2_-induced apoptosis, oxidative stress, and inflammation in NP cells	Tang et al. (2018)
	Quinazoline	Dephosphorylate NF-κB/MAPKs signaling molecules such as IKKβ, IκBα, p65, ERK, JNK, and p38	Inhibit IL-1β-induced oxidative stress and inflammation in the NF-κB/MAPKs signaling pathway, promote cell proliferation, and prevent NP cell degeneration	Chen et al. (2020)
	Leptin	Regulate the JNK/P38-MAPK/ERK and JAK2/STAT3 pathways to upregulate the mRNA expression levels of MMP-1, MMP-13, ADAMTS-4, ADAMTS-5, and COL2A1	Promote the expression of catabolic genes in rat NP cells	Miao and Zhang (2015)
	Crocin	Inhibit JNK phosphorylation	Inhibit LPS-induced inflammatory response and reduce ECM catabolism	Li K. et al. (2015)

## 6 Conclusion and future prospects

This study provides an overview of the pathogenic mechanism of IDD and discusses the current research status of the role of the JNK signaling pathway in IDD. The JNK signaling pathway regulates IDD by influencing various cellular mechanisms such as PCD, cell aging, inflammation, proliferation, ECM degradation, and OS. Furthermore, upstream and downstream signaling molecules of the JNK pathway can impact physiological processes like intervertebral disc PCD, inflammation, and ECM degradation by modulating the JNK pathway. However, there is limited literature on the involvement of cell aging, OS, and ferroptosis in JNK and IDD, and further research is needed to explore the relationship between IVD cell pyroptosis, necroptosis, pan-apoptosis, and the JNK signaling pathway in IDD. The JNK pathway interacts with multiple cellular mechanisms in IDD and can crosstalk with other signaling pathways, presenting a challenge in achieving precise targeted treatment of IDD without disrupting the normal IVD cell function. This study discusses various drugs and natural compounds that regulate IDD via the JNK signaling pathway. However, specific regulatory targets and mechanisms of the JNK pathway in IDD remain unclear. This study encourages researchers to further investigate the JNK signaling pathway in IDD to gain a better understanding of the molecular pathogenesis of IDD, but related research is scarce, and mostly non-clinical. Therefore, there is no clear evidence that the above-mentioned drugs or natural compounds can be applied in humans, and their effectiveness and safety remain to be studied. This will enhance the development of targeted therapies for IDD, using JNK as a therapeutic target, leading to more effective treatment strategies and innovative approaches.
